# First molecular subtyping and phylogeny of *Blastocystis* sp. isolated from domestic and synanthropic animals (dogs, cats and brown rats) in southern Iran

**DOI:** 10.1186/s13071-020-04225-9

**Published:** 2020-07-22

**Authors:** Iraj Mohammadpour, Farzaneh Bozorg-Ghalati, Alessia Libera Gazzonis, Maria Teresa Manfredi, Mohammad Hossein Motazedian, Niloofar Mohammadpour

**Affiliations:** 1 Department of Medical Parasitology and Mycology, School of Medicine, Shiraz University of Medical Sciences, Shiraz, Iran; 2 Department of Molecular Medicine, School of Advanced Medical Sciences and Technologies, Shiraz University of Medical Sciences, Shiraz, Iran; 3 Department of Veterinary Medicine, Università degli Studi di Milano, Milan, Italy; 4 Department of Medical Laboratory Sciences, Zeinab Hospital, Shiraz University of Medical Sciences, Shiraz, Iran

**Keywords:** *Blastocystis*, Subtyping, Phylogenetic analysis, *Canis lupus familiaris*, *Felis catus domesticus*, *Rattus norvegicus*, Iran

## Abstract

**Background:**

*Blastocystis* sp. is a common intestinal protist that infects humans and many animals globally. Thus far, 22 subtypes (STs) have been identified in mammalian and avian hosts. Since various STs are common to humans and animals, it was suggested that some human infections might arise from zoonotic transmission. Therefore, the aim of this study was to assess the presence of *Blastocystis* sp. in domestic (dogs and cats) and synanthropic animals (rats) of Fars Province, Iran, and to genetically characterize the samples.

**Methods:**

A total of 400 fresh faecal samples from 154 dogs, 119 cats, and 127 rats were inspected by direct microscopy, Wheatley’s trichrome staining, *in vitro* culture, and *18S* rRNA gene nested-PCR. Finally, sequencing and phylogenetic analyses were performed.

**Results:**

Out of 400 samples, 47 (11.8%) and 61 (15.3%) samples were detected as positive by direct wet mount and culture, respectively. Molecular analysis detected a larger number of positive samples (*n* = 70, 17.5%): nested-PCR showed that 29 (18.8%) dogs, 21 (17.7%) cats, and 20 (15.8%) rats were infected by *Blastocystis* sp. Sequence analysis of positive samples indicated the presence of zoonotic STs in all investigated host species. Specifically, ST2 (allele 9), ST3 (allele 34), ST4 (allele 94), ST7 (allele 99), ST8 (allele 21), and ST10 (allele 152) were detected in dogs; ST1 (allele 2), ST3 (allele 34), ST4 (allele 94), ST10 (allele 152), and ST14 (allele 159) were detected in cats; and ST1 (allele 2), ST3 (allele 34), and ST4 (allele 92) were detected in rats.

**Conclusions:**

Our data suggest that domestic dogs and cats can serve as possible reservoirs for in-contact humans, especially those who handle shelter-resident and client-owned animals. Moreover, rats as synanthropic animals can function as a potential source of human infections. Conversely, humans can act as a source of infections to animals. These results should be reinforced in future molecular epidemiological studies.
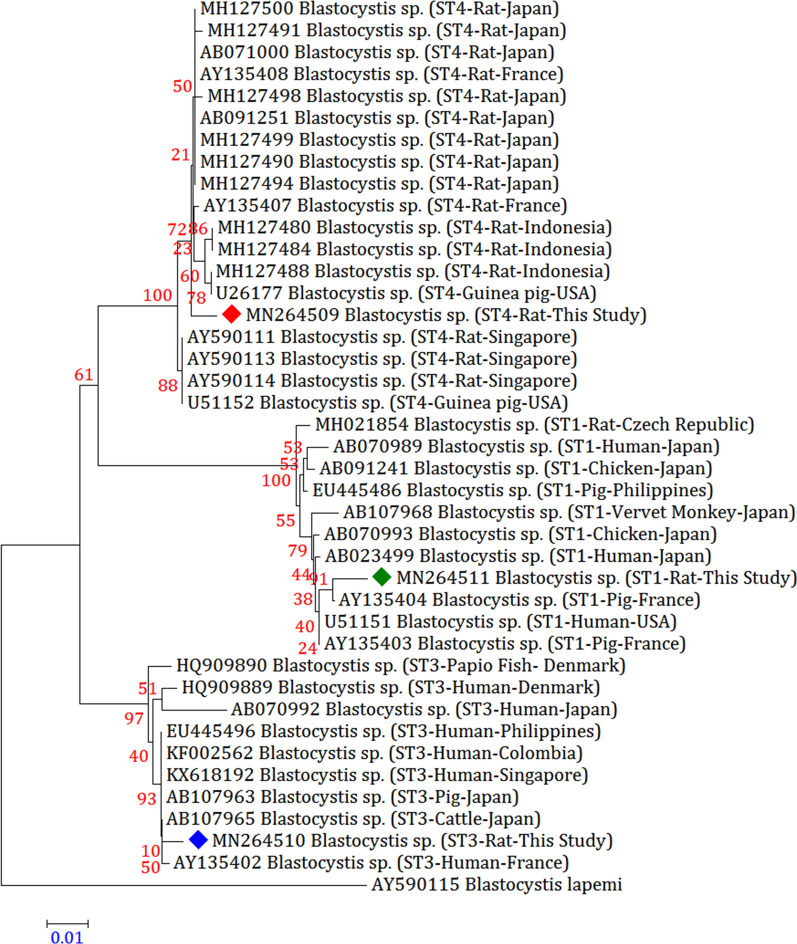

## Background

*Blastocystis* sp. is an anaerobic eukaryotic protist belonging to the phylum Stramenopiles. It is isolated from the lumen of humans and animals and has four different morphological forms (central-body, granular, amoeboid and cystic) [1]. Binary fission, budding, and plasmotomy are the forms of reproduction observed in *Blastocystis* sp. [2]. *Blastocystis* sp. has a global distribution, with a recently reported prevalence of up to 100% in humans [3]. It is believed that *Blastocystis* sp. is transmitted *via* the faecal-oral route, water, food, and direct person-to-person contact [1, 4]. There is supporting evidence that zoonotic transmission of *Blastocystis* sp. from animals to humans living in a community-based environment may occur *via* close contact with animal enclosures [5–7].

In recent years, several studies have reported that *Blastocystis* sp. is a causative agent of diarrhoea, abdominal pain, flatulence, irritable bowel syndrome, inflammatory bowel disease, and urticaria [8, 9]. However, its pathogenicity is still a topic of some debate, as asymptomatic carriage is common [10, 11]. Considerable evidence suggests that *Blastocystis* sp. is capable of producing cysteine proteinases that interpose interleukin-8 release from enterocytes, impelling enterocytes apoptosis and augmenting gut permeability while also potentially evading recognition by Toll-like receptors [12, 13].

*Blastocystis* sp. has been shown to exhibit extensive genetic diversity [14, 15]. Hitherto, 22 different subtypes (STs) of *Blastocystis* sp., consisting of ST1 to ST17, ST21, and ST23 to ST26 have been described in humans and a variety of animals based on polymorphisms in *18S* rRNA gene sequences [16]. Nevertheless, the prevalence of the different subtypes varies among countries and among regions within the same country. ST1 to ST9 and ST12 have been isolated from humans, with ST3 being the most frequent subtype, and all except ST9 have also been reported in animals [17, 18]. ST5 is prevalently isolated from livestock, ST6 and ST7 from birds, and ST8 from non-human primates (NHPs) [19–22]. The proof that ST5 to ST8 have only been fortuitously discovered in humans has been interpreted as suggestive of zoonotic transmission [19–22]. ST10 to ST17, ST21, and ST23 to ST26 have been recorded only in non-human hosts thus far [14–16].

A substantial number of animal species have been examined for the identification of *Blastocystis* sp. [5, 14, 20–23]. However, more studies are required to characterize the zoonotic transmission of this parasite and to identify whether other subtypes of *Blastocystis* sp. exist. The analysis of DNA extracted directly from stool samples is considered to be highly sensitive, providing the means for genotyping and subtyping [24, 25]. Diagnostic real-time PCR has been recently introduced; however, so far it has not been used for screening [26].

*Blastocystis* sp. has been reported to have prevalence rates of 2‒47% in livestock and 8‒67% in captive animals from zoological gardens [14, 20–22, 27]. Presently, an expanding interest in *Blastocystis* sp. research fortifies the necessity for investigations of this parasite in areas where it has not yet been assessed. In Iran, molecular studies were performed in relation to human infection [28]. Concerning animals, *Blastocystis* sp. was previously genotyped from hooded crows and pigeons [29], cattle [30] and wild boars [31], while there are no molecular studies regarding the occurrence of *Blastocystis* sp. in animals living in close contact with humans, such as pets and synanthropic animals. Therefore, the aim of this study was to assess the presence of *Blastocystis* sp. in dogs, cats and rats of Fars Province, southern Iran, and to genetically characterize the samples by *18S* rRNA gene sequencing. To the best of our knowledge, this is the first and largest epidemiological study executed on these animals in Iran.

## Methods

### Sample collection

For the present cross-sectional study, 400 fresh faecal samples from 154 dogs (*Canis lupus familiaris*), 119 cats (*Felis catus domesticus*), and 127 brown rats (*Rattus norvegicus*) were collected from December 2016 to October 2018. The study was performed on dogs and cats regardless of their race, age or sex.

Dogs’ fresh faecal samples were collected individually per-rectum under an anaesthesia regime during a trap, neuter, and release (TNR) sterilization programme to restrain stray dog populations controlled by the Fars Veterinary Administration.

After consulting with the vector control unit of the Fars Veterinary Administration, searching and identifying the active colonies of rodents in different places of Fars Province were carried out and wild rodents were trapped. Cats and brown rats were lured in the same spatiotemporal location using Sherman baited cage-traps with tinned fish and roasted walnuts, respectively. Wire cage-traps were placed at rodents’ burrows entrances in gardens and grasslands around the residential houses in different cities and counties of Fars Province. Traps were set in the evening and were checked the next day in the morning. Faeces of captive animals was collected from their cages to minimize contamination using disposable spoons, stored in tagged polystyrene flasks, and transported as early as possible to the head laboratory. Gross macroscopic examination of faecal samples was done to check for consistency. The consistencies of the collected faecal specimens were formed and soft. Every time a mean of 10 traps was used for screening and processing.

### Microscopic examination

Samples were processed within 2‒4 h after collection. Faecal smears were prepared and first examined by direct microscopy using both a saline and iodine wet mount preparation. In addition, faecal smears were stained with Wheatley’s trichrome stain.

### Faecal culture

*In vitro* culture was concurrently carried out for all stool samples using Jones’ medium supplemented with 10% heat-inactivated horse serum (Gibco, Frankfurt, Germany), 10% Pen-Strep (1000 IU/ml and 500 μg/ml) and powdered rice-starch. Nearly 200 mg stool specimens were inoculated into 5 ml screw-cap tubes containing 3 ml of Jones’ medium using a sterile disposable applicator stick. The culture tubes were incubated at 37 °C for 72 h [32]. The presence of any of the 4 morphologies of *Blastocystis* sp. was observed daily by placing one drop of culture product onto SAF-coated coverslips and staining with Wheatley’s trichrome stain. Positive samples were subcultured on LYSGM medium supplemented with 10% heat-inactivated horse serum (Gibco) for mass cultivation [32].

### DNA extraction

To lessen impediments from raw fibres and impurities, the faecal specimens were sieved and washed 3 times with distilled water by centrifugation at 1500×*g* for 10 min. Total genomic DNA of *Blastocystis* sp. was extracted using a QIAamp^®^ Fast DNA Stool Mini Kit (Qiagen, Hilden, Germany) according to the manufacturer’s instructions. DNA quality was verified by NanoDrop^®^ (Thermo Fisher Scientific, Carlsbad, CA, USA) measurements. The final DNA was eluted in 100 μl of AE buffer to increase its concentration and stored at − 20 °C until use.

### Nested-PCR amplification

Amplification of a 1100-bp fragment of the *Blastocystis* sp. *18S* rRNA gene was carried out using a two-step nested-PCR. This method was chosen since it provides a large fragment for precise sequencing and phylogenetic analysis, and as it is a pan-*Blastocystis* sp. technique, it permits the identification of known and unknown subtypes of *Blastocystis* sp. [33].

The primers RD3 (5′-GGG ATC CTG ATC CTT CCG CAG GTT CAC CTA C-3′); RD5 (5′-GGA AGC TTA TCT GGT TGA TCC TGC CAG TA-3′); BlF (5′-GGA GGT AGT GAC AAT AAA TC-3′); and BlR (5′-CGT TCA TGA TGA ACA ATT AC-3′) were used for the first and second rounds of the nested-PCR. The first PCR reactions were performed in 25 μl reaction mixtures containing 1× PCR buffer (10 mM Tris-HCl, pH 9, 50 mM KCl), 1.5 mM MgCl_2_, 0.2 mM of each dNTPs (Roche, Alameda, CA, USA), 1.25 U Platinum^®^*Taq* High-Fidelity DNA polymerase (Invitrogen, Groningen, the Netherlands), 40 µg/ml of BSA, 10 pM of each forward and reverse primers, and 2 μl genomic DNA.

The PCR conditions consisted of pre-denaturation at 95 °C for 5 min, 40 cycles of denaturation at 95 °C for 30 s, annealing at 65 °C for 45 s, extension at 72 °C for 1 min and a final extension at 72 °C for 10 min using an Eppendorf Mastercycler Gradient PCR machine (Eppendorf, Hamburg, Germany). The second PCR cycle was carried out with similar reaction systems except using the PCR products of the first cycle as the template, and 54 °C as the annealing temperature. Electrophoresis was performed by adding 2 µl of the PCR products and a 100-bp molecular marker (GenScript, Tokyo, Japan) loaded on 1.5% agarose gels (AddGene, Watertown, MA, USA) in TBE buffer and staining with SYBR^®^ Safe DNA Gel Stain (Thermo Fisher Scientific) for 1 h at 90 V. Bands were observed by UV light and photographed (Uvitec, Cambridge, UK). Positive and negative controls were included in every PCR run.

### Sequencing and phylogenetic analysis

Bands of the expected size were excised from the agarose gel and purified using a QIAquick^®^ Gel Extraction Kit (Qiagen) according to the manufacturer’s instructions. Purified products were sequenced in both directions using nest-2 primers at Roche Molecular Diagnostics (Roche, Mannheim, Germany) by capillary electrophoresis using a Big Dye^TM^ Terminator Cycle Sequencing Kit (Applied Biosystems, Foster City, CA, USA) in an ABI PRISM^®^ 3730 automated sequencer.

Raw sequencing data in both the forward and reverse directions were checked using Chromas 2.6.6 (Technelysium, Brisbane, Australia). Special attention was paid to the double peaks (indicative of mixed infections by different subtypes or alleles) and the accuracy of the nucleotides was guaranteed. Multiple sequence alignment was performed using the MUSCLE algorithm of MEGA-X [34]. The consensus sequences were then compared with homologous sequences available in the GenBank database using the nBLAST program. The sequences were assembled and edited with BioEdit (v.7.2.6; https://www.bioedit.com). Subtypes were determined by exact match or an identity ≥ 98% against all known *Blastocystis* sp. subtypes, with a query coverage of ≥ 98%.

All established sequences were submitted to the multilocus sequence typing (MLST) database (https://pubmlst.org/blastocystis/) for subtype confirmation and relevant allele identification.

Two molecular phylogenetic trees (one for dogs and cats, and another for rats) were constructed with the Neighbour-Joining (NJ) method, and genetic distances were calculated with the Maximum Composite Likelihood model in MEGA-X [34]. Bootstrap analysis (with 1000 replicates) was carried out to define the robustness of the findings. *Proteromonas lacertae* and *Blastocystis lapemi* were used as outgroups for the two analyses.

### Statistical analysis

The agreement between microscopy, culture, and PCR results was measured using the Cohen’s kappa coefficient (κ) (GraphPad Prism^®^, Melbourne, Australia) and results were interpreted as follows: no agreement (κ ≤ 0); slight agreement (0.01 < κ < 0.2); fair agreement (0.21 < κ < 0.4); moderate agreement (0.41 < κ < 0.6); substantial agreement (0.61 < κ < 0.8); and almost perfect agreement (0.81 < κ ≤ 1).

Fisher’s exact test was used to compare the frequency of *Blastocystis* sp. carriage between dogs, cats, and rats (based on molecular results). The significance level was established at *P*  < 0.05.

## Results

### Infection rates, subtyping and allele analysis

Overall, 400 faecal samples were collected in the current study from 154 dogs, 119 cats, and 127 rats and investigated for *Blastocystis* sp. Out of 400 faecal samples, 47 (11.8%) samples were shown to be positive by direct wet mount and Wheatley’s trichrome staining examination. The most common finding was the central-body form of the protozoan (Additional file 1: Figure S1). Among 400 stool samples, 61 (15.3%) were found to be positive by *in vitro* cultivation in Jones’ medium. The most common forms of *Blastocystis* sp. in Jones’ medium were the central-body and granular forms. κ-values of 0.461, 0.706 and 0.864 were appraised between microscopy, culture, and PCR (Table 1).

**Table 1:** The estimated κ-value between microscopy, culture, and PCR for identification of *Blastocystis* sp

Method	κ-value	Degree of agreement
Microscopy	0.461	Moderate
Culture	0.706	Substantial
PCR	0.864	Perfect

Nested-PCR was carried out for amplification of a 1100-bp band (Additional file 2: Figure S2). Overall, 29 faecal samples from dogs (18.8%), 21 faecal samples from cats (17.7%), and 20 faecal samples from rats (15.8%) were detected as positive by nested-PCR (Table 2). No statistically significant differences in prevalence values were recorded among the three species studied (*P* > 0.05). All positive samples were sequenced successfully. The partial sequences of the *18S* rRNA gene of *Blastocystis* sp. obtained in this study were deposited in the GenBank database under accession numbers MN264509–MN264522.

**Table 2:** Percentage of infection and subtype/allele distributions of *Blastocystis* sp. detected in dogs, cats, and rats in Fars Province, Iran

Host species	Positive/examined (%)	Subtype/alleles (*n*)	GenBank ID
Brown rat (*Rattus norvegicus*)	20/127 (15.8)	ST1/ allele 2 (*n* = 4)	MN264511
		ST3/allele 34 (*n* = 4)	MN264510
		ST4/allele 92 (*n* = 12)	MN264509
Cat (*Felis catus domesticus*)	21/119 (17.7)	ST1/allele 2 (*n* = 5)	MN264518
		ST3/allele 34 (*n* = 7)	MN264519
		ST4/allele 94 (*n* = 4)	MN264520
		ST10/allele 152 (*n* = 3)	MN264521
		ST14/allele 159 (*n* = 2)	MN264522
Dog (*Canis lupus familiaris*)	29/154 (18.8)	ST2/allele 9 (*n* = 8)	MN264512
		ST3/allele 34 (*n* = 11)	MN264513
		ST4/allele 94 (*n* = 3)	MN264514
		ST7/allele 99 (*n* = 3)	MN264516
		ST8/allele 21 (*n* = 2)	MN264515
		ST10/allele 152 (*n* = 2)	MN264517

Among the 70 *Blastocystis*-positive samples, 8 subtypes were detected: 6 zoonotic STs (ST1–ST4, ST7, and ST8), ST10, and ST14. Dogs were the hosts to the widest range of STs, followed by cats and rats. Remarkably, 6 subtypes were found in the dogs, including ST2 (allele 9), ST3 (allele 34), ST4 (allele 94), ST7 (allele 99), ST8 (allele 21), and ST10 (allele 152). Meanwhile, 5 subtypes were detected in the cats, comprising ST1 (allele 2), ST3 (allele 34), ST4 (allele 94), ST10 (allele 152), and ST14 (allele 159). In addition, 3 subtypes were found in the rats, including ST4 (allele 92), ST3 (allele 34), and ST1 (allele 2) (Table 2). No mixed infections involving different STs of the protist were identified. No intra-subtype genetic heterogeneity was observed within detected subtypes.

### Phylogenetic analysis

A total of 14 representative sequences were obtained from 70 *Blastocystis* sp. isolates in the present study (Table 2). The sequences obtained in this survey shared high identity with the *Blastocystis* sp. sequences registered in GenBank.

**Fig. 1:**
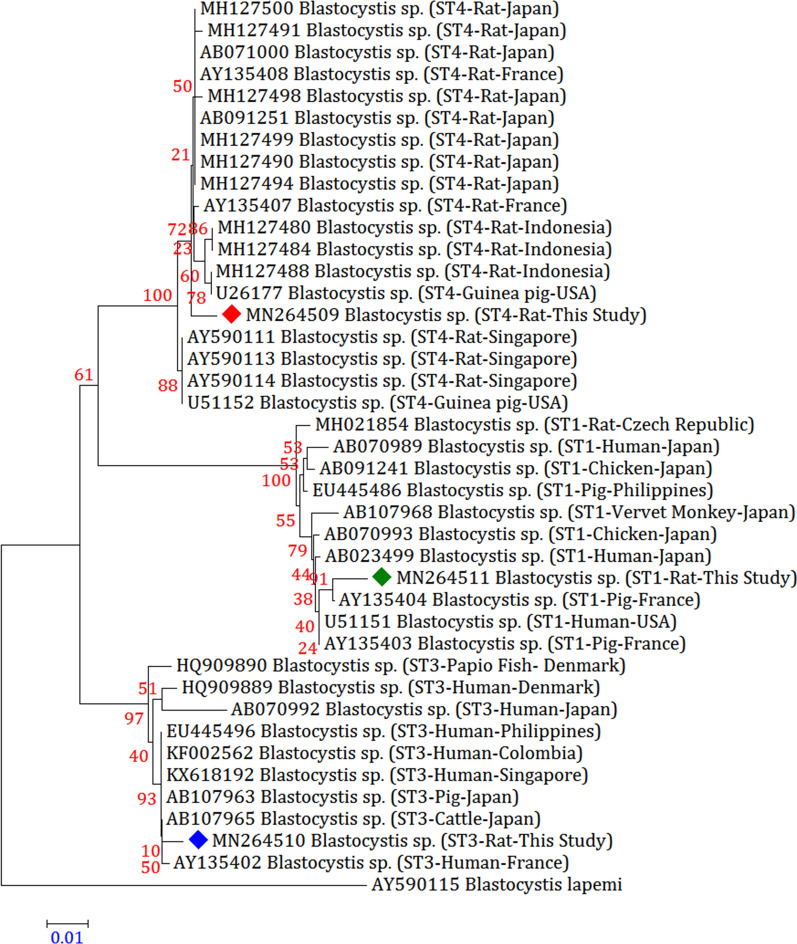
Molecular phylogenetic relationships between various *Blastocystis* sp. samples isolated from rats as inferred by the Neighbour-Joining tree based on the *18S* rRNA gene. The numbers on branches are percentage bootstrap values of 1000 replicates. The evolutionary distances between sequences were computed using the Maximum Composite Likelihood method. The scale-bar indicates an evolutionary distance of 0.01 nucleotides per position in the sequence. The reference sequence accession numbers are shown. Evolutionary analyses were conducted in MEGA-X. *Blastocystis lapemi* was used as the outgroup

**Fig. 2:**
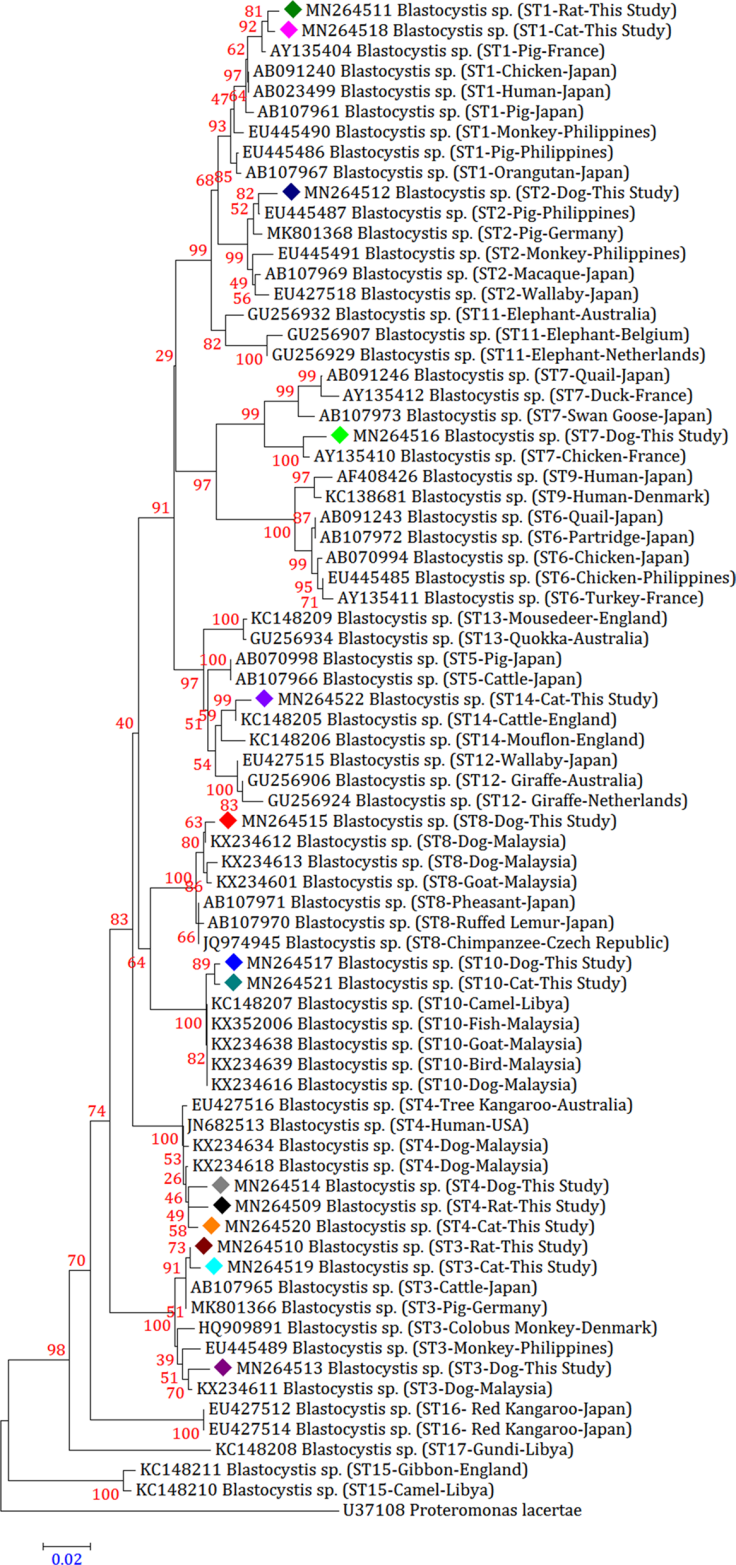
Molecular phylogenetic relationships between various *Blastocystis* sp. samples isolated from cats and dogs as inferred by the Neighbour-Joining tree based on the *18S* rRNA gene. The numbers on branches are percentage bootstrap values of 1000 replicates. The evolutionary distances between sequences were computed using the Maximum Composite Likelihood method. The scale-bar indicates an evolutionary distance of 0.02 nucleotides per position in the sequence. The reference sequence accession numbers are shown. Evolutionary analyses were conducted in MEGA-X. *Proteromonas lacertae* was used as the outgroup

When the phylogenetic analysis was executed, in contrast to the 17 reference subtype sequences in GenBank, we procured a precise discrimination of *Blastocystis* sp. subtypes compatible with that established by BLAST queries. Because our study confirmed that ST4 was the most dominant subtype in southern Iranian rodent population, so two phylogenetic trees were constructed to compare this subtype with other rat-derived ST4. All sequences obtained from rats, cats, and dogs were closely related to animal- or human-derived sequences in GenBank and clustered together (Figs. 1 and 2).

Inter-subtype genetic polymorphism was discerned between detected subtypes and reference subtypes registered in GenBank. According to homology analysis, the two ST1 sequences derived from rats and cats (MN264511 and MN264518, respectively) were the same and were 99.36% and 98.91% similar to those from pigs in France (AY135404) and from humans in Japan (AB023499). The only ST2 sequence obtained from dogs (MN264512) shared 99.36% and 98.27% identity with that from pigs in the Philippines (EU445487) and from monkeys in Japan (AB107969). Among the three ST3 sequences, one rat-derived and one cat-derived *Blastocystis* sp. isolate produced the same sequences (MN264510 and MN264519, respectively), with these sequences being 99.45%, 99.36%, and 99.36% similar to those in cattle in Japan (AB107965), humans in the Philippines (EU445496), and pigs in Germany (MK801366), respectively. The remaining dog-derived ST3 sequence (MN264513) was 99.27% similar to that from dogs in Malaysia (KX234611).

Among the three ST4 isolates, three different sequences were obtained. The rat-derived ST4 sequence (MN264509) was 99.36% and 99.27% similar to that from rats in France (AY135408) and from rats in Japan (AB071000 and MH127499), respectively. The cat-derived ST4 sequence (MN264520) shared 99.36% identity with that from humans in the USA (JN682513) and from rats in Indonesia (MH127488). The remaining dog-derived ST4 sequence (MN264514) was 99.36% similar to that from dogs in Malaysia (KX234618). The only dog-derived ST7 sequence (MN264516) was 99.36% similar to that from chickens in France (AY135410). In addition, the only dog-derived ST8 sequence (MN264515) was 99.36% similar to that from dogs in Malaysia (KX234612).

Among the two ST10 isolates, two different sequences were obtained. The dog-derived ST10 sequence (MN264517) shared 99.36% identity with that from dogs in Malaysia (KX234616). The cat-derived ST10 sequence (MN264521) was 99.36% similar to that from camels in Libya (KC148207) and from goats in Malaysia (KX234638). The only cat-derived ST14 sequence (MN264522) shared 99.27% identity with that from cattle in England (KC148205).

## Discussion

*Blastocystis* sp. is an enteric protist with a universal distribution. It is assumed that multiple zoonotic isolates of *Blastocystis* sp. exist with regular animal-to-human and human-to-animal transmission and as a large potential reservoir in animals for infections in humans [4, 35–37]. Recent genomic data revealed putative virulence factors and illustrated deleterious clinical outcomes of *Blastocystis* sp. on the intestinal barrier, leading to conceivable models of pathogenesis [10–13, 38].

*Blastocystis* sp. has been secluded from a large spectrum of animals, such as birds, pigs, cattle, sheep, goats, wild carnivores, NHPs, and less frequently rodents, amphibians, reptiles, and insects [21–23, 39–41]. *Blastocystis* sp. has cryptic host specificity and is heeded potential zoonotic protozoa since infections in humans have been connected with contact with various mammals and birds [21, 42, 43]. Furthermore, successful experimental infections of rats after oral administration of ST1, ST3, and ST4 purified cysts isolated from human stool samples confirmed the transmission of this protist between human and animal hosts [44, 45]. A higher risk of *Blastocystis* sp. infection was found in people with close animal contact, supporting the hypothesis of transmission from animals to humans [5, 6, 46, 47].

In a territory where *Blastocystis* sp. is largely present in humans, there is a necessity to determine the load of this infection in animals and to identify the reservoirs or sources of infection in the community. In Iran, several related studies have been accomplished on the human population [28], while few surveys have been performed for domestic and wild animals [29–31]. To the best of our knowledge, our study represents the first inspection of and report on the prevalence of *Blastocystis* sp. subtypes isolated from domestic and synanthropic animals (dogs, cats and brown rats) in Fars Province, Iran. Three diagnostic investigations were conducted in parallel on the 400 faecal samples included in the present study: direct microscopy; *in vitro* culture; and nested-PCR. Studies have shown that PCR is a valid technique with high sensitivity, specificity, reliability and reproducibility [24]. In the present study, nested-PCR allowed us to identify the largest number of positive samples. Previous studies carried out in animals in Iran showed higher infection rates in hooded crows and pigeons (42.9% and 44.4%, respectively) [29] and in wild boars (25.0%) [31], although a lower infection rate was reported in cattle (9.6%) [30].

Globally, epidemiological studies conducted on dogs and cats to investigate their potential role as natural reservoirs of human *Blastocystis* sp. infection revealed disparate prevalence values. Surveys targeting sheltered and household canine and feline populations did not demonstrate the presence of *Blastocystis* sp. in Japan and Spain [48, 49], while infection rates in the range of 9.7‒37% have been documented in shelter dogs in the USA [7], in stray dogs in India [33], in dogs housed in Italian rescue shelters [50], and in symptomatic dogs and cats attending a veterinary clinic in Chile [51].

Concerning brown rats, the recorded prevalence of 15.8% was in line with that reported from previous surveys on rodents, ranging from 8.4% to 45.9% [52, 53]. The large number of positive brown rats, synanthropic animals that share spaces with humans and contaminate the environment with faeces and urine, strengthens their role as potential reservoir hosts for *Blastocystis* sp.

Furthermore, this study is the first to assess the subtype diversity of *Blastocystis* sp. based on the *18S* rRNA gene in dogs, cats and rats in Fars Province, Iran. Currently, human *Blastocystis* sp. isolates are classified as ST1-ST9 and ST12, and all except ST9 have also been identified in other animals [17, 18, 25, 39]. In particular, ST1 to ST4 are among the most shared subtypes in humans and have been associated with human disease, having low host specificity and probable zoonotic connotation [54, 55].

In our study, 8 subtypes were identified: ST1–ST4; ST7; ST8; ST10; and ST14. It is noteworthy that the three investigated species harboured not only the same STs, but also the same alleles of each subtype, indicating a possible common environmental source of infection. ST1, detected in the present study in cats and brown rats, was previously reported in captive wild animals, NHPs, sheep, goats, pigs, water voles, marsupials and birds in various locations [21–23, 27, 56–61]. ST2, recorded in eight dogs, is considered the dominant subtype in NHPs [61]. The zoonotic potential of NHP isolates was previously investigated, in which ST2 isolates with identical sequences were found in rhesus monkeys and children living in the same area of Nepal [46]. Moreover, ST1 and ST2 have been found in zookeepers and five primate species in Australia [5]. ST3 is the *Blastocystis* sp. subtype with the highest prevalence in humans worldwide and is probably a human species-specific subtype [35, 54, 62]. The finding of ST3 in the three investigated species supported the potential role of domestic and synanthropic animals in human *Blastocystis* sp. infections.

In the present study, ST1, ST3, and ST4 were detected in brown rats. ST4 was the most prevalent, and it was also found in cats and dogs. Generally, rodents have been shown to be the chief animal reservoir of ST4, which is an ancestry with recent entry into the human population [63, 64]. ST4 has been the most prevalent subtype found in Danish *Blastocystis*-positive patients presenting with acute diarrhoea [55]. Interestingly, ST4 is reported at low prevalence rates in asymptomatic, apparently healthy individuals in Spain [49]. Most epidemiological studies describing ST4 in human populations depict allele 42, allele 91, and allele 133 of this subtype [49, 65]. However, the ST4 genetic variant reported in rats here is allele 92. This is an interesting point that should be further investigated. In addition, ST4 has been found in NHPs, giraffes, kangaroos, dogs, pigs and ostriches [20, 33, 57, 61, 63]. Moreover, ST3 and ST4 were identified in cockroaches [41, 61, 66].

In the present survey, ST7 and ST8 were also detected in dogs. ST7 is one of the most frequent subtypes in birds and is principally considered an avian subtype [57, 62]. In addition to birds, ST7 has been reported in pigs, goats and dogs [22, 33, 56, 58]. Furthermore, ST8 has been reported more prevalently in NHP handlers, suggesting a zoonotic scatter from primates to their handlers [61, 63]. Recently, ST8 was identified in pigs and marsupials [20, 61].

ST10, detected in the present study both in dogs and cats, has been reported exclusively in non-human hosts, including wild animals and livestock (cattle, yaks, camels, pigs, sheep and goats) in Libya, UK, China, Malaysia, Japan and the UAE [15, 56, 58, 59, 67–70]. ST10 was also detected in cats from the USA and dogs from France [7, 71].

Finally, we detected ST14 in two cats. ST14 is similar to ST10 in terms of host range, infecting some common livestock and some artiodactyls [57, 68]. Recently, ST14 was isolated from hooded crows and pigeons in Iran [29].

In dogs, ST1 to ST3 have been identified in Italy [50], France [71], Australia [72], and the Philippines [73], suggesting that dogs could be involved in the transmission of *Blastocystis* sp. to humans. Dogs are proficient in shedding possibly zoonotic subtypes and may act as incidental zoonotic reservoirs for infection. Particularly, direct evidence of zoonotic transmission has been granted by other surveys examining human and canine/feline populations living in the same spatiotemporal setting. In a prior study, eight dogs in close contact with 11 symptomatic family members infected by *Blastocystis* sp. were all positive for the parasite and harboured at least one subtype shared with each of the corresponding patients, suggesting that the source of infection of the owners was their household dogs [72]. *Blastocystis* ST2 to ST5 were concurrently found in people and domestic dogs living in an urban community in the Philippines [73]. Likewise, ST5 was identified in a villager and a dog both living in a village in Thailand signifying the probability of dogs as sources of zoonotic transmission of *Blastocystis* sp. [74]. Moreover, all *Blastocystis* sp. isolated from dogs in India and Indonesia were characterized as zoonotic subtypes [33, 75]. Contrarily, a recent study demonstrate that pet dogs and cats play a tiny role as natural reservoirs of human *Blastocystis* sp. infection in Spain [49].

Specifically, stray dogs, which generally live in areas with poor sanitation and hygiene, were identified to be at greater risk of transferring *Blastocystis* sp. than domestic dogs [75]. Stray dogs in India carried a diverse range of *Blastocystis* sp. subtypes, including ST1 and ST4 to ST6, while Australian and Cambodian dogs harboured only ST1 and ST2, respectively [33]. Furthermore, ST1 was found in shelter-resident dogs and cats in the Pacific Northwest of the USA, emphasizing the potential role of stray dogs and cats as the origin of human *Blastocystis* sp. infection [7].

People who have close contact with dogs and cats for occupational or leisure reasons are at a high risk of acquiring *Blastocystis* sp. infection. Faced with the fact that coprophagia is a common instinct in dogs, the greater prevalence and diversity of subtypes found in dogs could be ascribed to their increased environmental exposure to faecal content from humans, cattle, sheep, goats and birds. Meanwhile, *Blastocystis* sp. in animal faeces can enter creeks and rivers by surface run-off after extreme rainfall, which allows water contamination downstream and broad geographical dispersion of *Blastocystis* sp. The environmental pollution level, size of cysts, resistance of cysts to water treatment methods such as chlorination, and poor elimination of cysts during the filtration process permit the zoonotic transmission of *Blastocystis* sp. [76].

## Conclusions

The findings of this study showed that dogs, cats, and rats in Fars Province, Iran are shedding *Blastocystis* sp. Eight subtypes were characterized, with subtype overlaps between animals. Dogs, cats and rats are presumed to be part of the transmission dynamics of this infection in Fars Province. Although the role of these animals as a possible natural reservoir of *Blastocystis* sp. remains obscure, it seems that these animals could represent possible reservoirs of zoonotic transmission of *Blastocystis* sp. These data also accentuate the significance of screening other hosts of *Blastocystis* sp. in order to fully characterize the epidemiology of this protist. Given that extensive genetic diversity exists within *Blastocystis* sp. subtypes, future molecular characterization and comparisons of dog, cat, rat, human and other mammalian *Blastocystis* sp. subtypes using multilocus sequence typing (MLST) performed within communities where *Blastocystis* sp. is endemic in dogs, cats and humans will ideally shed further light on their role as natural hosts for infection. This epidemiological cohort study also furnishes essential information for taking preventive and control measures that should help lessen the risks of zoonotic transmission of *Blastocystis* sp. to humans.

## Supplementary information

**Additional file 1: Figure S1.** The central-body form of *Blastocystis* sp. stained with Wheatley’s trichrome stain (1000× magnification).

**Additional file 2: Figure S2.** Electrophoresis of PCR products of *Blastocystis* sp. DNA extracted from dog, cat, and rat faecal samples based on the *18S* rRNA gene. The nine lanes contain the products from the negative control (Lane 9), positive control (Lane 8), positive samples of *Blastocystis* sp. that shows a specific diagnostic band of the 1100-bp fragment (Lanes 1-7), and a molecular size marker (Lane MM).

## Data Availability

All data generated or analysed during this study are included in this published article. Sequences were submitted to the GenBank database under the accession numbers MN264509–MN264522.

## Abbreviations

ST: subtype
NHPs: non-human primates
TNR: trap, neuter, and release
SAF: sodium acetate-acetic acid-formalin
LYSGM: liver extract-yeast extract-serum-gastric mucin
BSA: bovine serum albumin
NJ: neighbour-joining
