# Supplementary figures and images for: First molecular subtyping and phylogeny of Blastocystis sp. isolated from domestic and synanthropic animals (dogs, cats and brown rats) in southern Iran
Source: Parasit Vectors. 2020 Jul 22;13:365. doi: 10.1186/s13071-020-04225-9 (PMC7374852; doi:10.1186/s13071-020-04225-9)

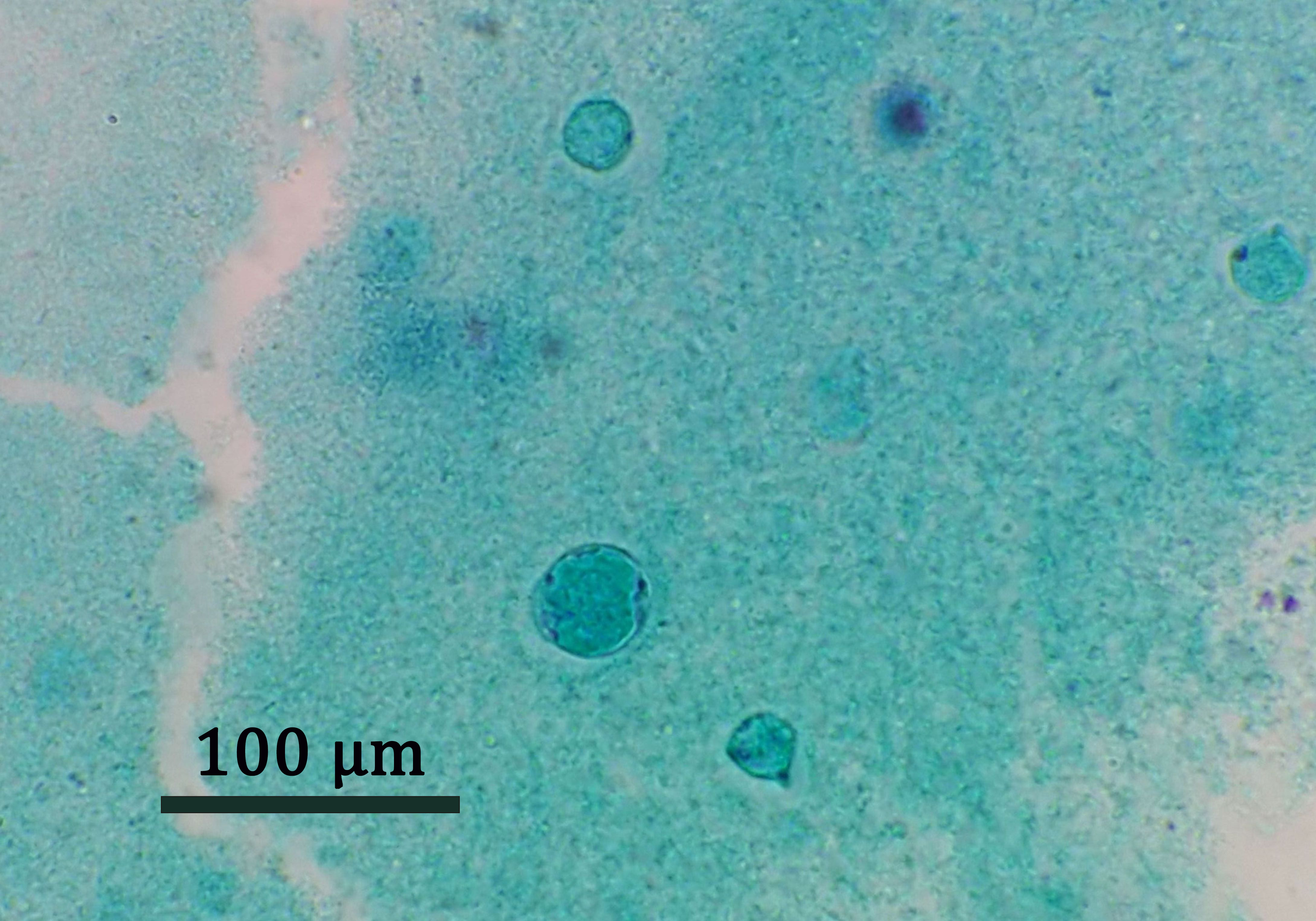

Supplement: Supplementary file 1 — Additional file 1: Figure S1. The central-body form of Blastocystis sp. stained with Wheatley’s trichrome stain (1000× magnification). [file 13071_2020_4225_MOESM1_ESM.png]

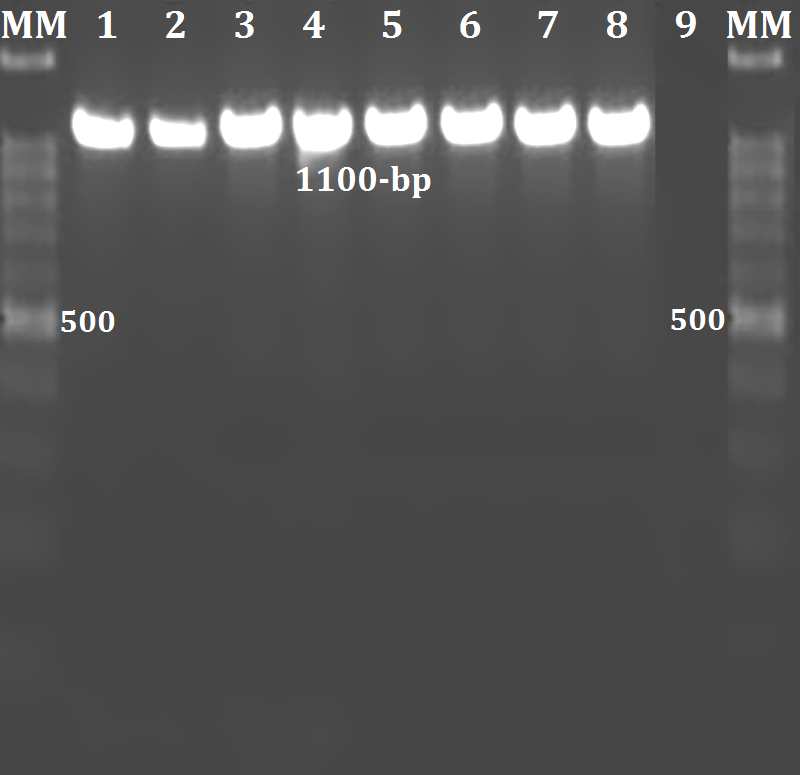

Supplement: Supplementary file 2 — Additional file 2: Figure S2. Electrophoresis of PCR products of Blastocystis sp. DNA extracted from dog, cat, and rat faecal samples based on the 18S rRNA gene. The nine lanes contain the products from the negative control (Lane 9), positive control (Lane 8), positive samples of Blastocystis sp. that shows a specific diagnostic band of the 1100-bp fragment (Lanes 1-7), and a molecular size marker (Lane MM). [file 13071_2020_4225_MOESM2_ESM.tif]
